# Criss-cross

**DOI:** 10.1007/s12471-018-1102-8

**Published:** 2018-03-08

**Authors:** R. van der Nagel, V. F. van Dijk

**Affiliations:** 0000 0004 0622 1269 grid.415960.f St. Antonius Ziekenhuis, Nieuwegein, The Netherlands

## Answer

**Fig. 1:**
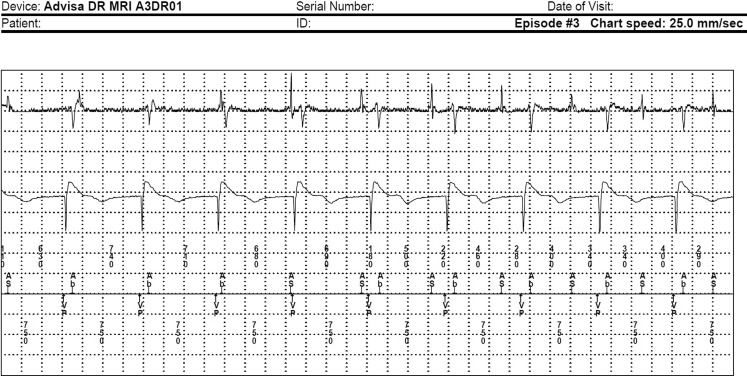
Intracardial electrogram of episode. Note the fixed rate of the ventricle (750 ms, 80 bpm) and the consistent far-field R wave. The sinus rhythm (680 ms, 88 bpm) is uninterrupted

An intracardial electrogram (iEGM) reveals the origin of this peculiar atrial patterning. First note the regular rhythm of the ventricle (750 ms, 80 beats per minute (bpm)) (Fig. 1). Since the patient has a known history of paroxysmal atrial fibrillation, and had an episode when we changed the parameters just before surgery, we opted to programme the device to the asynchronous mode VOO 80 during surgery. You can recognise the associated far-field R wave (FFRW) of the paced ventricular activation on the atrial iEGM channel as well. It is annotated as ‘ab’, as it is registered within the post-ventricular atrial blanking period. Because of the VOO mode, there is no absolute blanking and the sinus rhythm (680 ms, 88 bpm) is not affected by this anomaly. Dissociation between these two signals causes the pattern to rise.

**Fig. 2:**
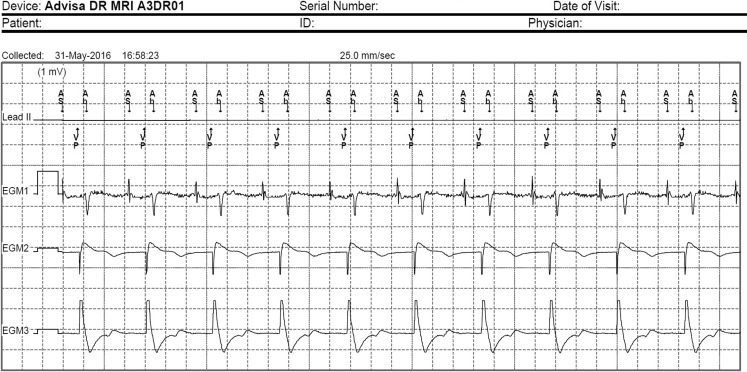
Sinus rhythm (AS) with paced ventricular response (VP). Note the far-field R wave, which is blanked absolutely by the device (Ab) in DDD mode

In normal settings (DDD 60-130), this phenomenon does not occur, as the FFRW is blanked by the device (Fig. 2). Note that both the intrinsic atrial rhythm (AS) and FFRW (Ab) are consonant with both signals found in the electrogram of the episode of atrial tachycardia/atrial fibrillation.

